# The Effect of a Cognitive Dual Task on Gait Parameters among Healthy Young Adults with Good and Poor Sleep Quality: A Cross-Sectional Analysis

**DOI:** 10.3390/jcm13092566

**Published:** 2024-04-27

**Authors:** Jood Dalbah, Shima A. Mohammad Zadeh, Meeyoung Kim

**Affiliations:** 1 Department of Physiotherapy, College of Health Sciences, University of Sharjah, Sharjah P.O. Box 27272, United Arab Emirates; u21106195@sharjah.ac.ae (J.D.); szadeh@sharjah.ac.ae (S.A.M.Z.); 2 Laboratory of Health Science & Nanophysiotherapy, Department of Physical Therapy, Graduate School, Yongin University, Yongin 17092, Republic of Korea; 3 Neuromusculoskeletal Rehabilitation Research Group, Research Institute of Medical and Health Sciences, University of Sharjah, Sharjah P.O. Box 27272, United Arab Emirates

**Keywords:** sleep quality, dual task, gait, velocity, cadence, stride length, step width

## Abstract

**Background**: Sleep quality is known to affect automatic and executive brain functions such as gait control and cognitive processing. This study aimed to investigate the effect of dual tasks on gait spatiotemporal parameters among young adults with good and poor sleep quality. **Methods**: In total, 65 young adults with a mean age of 21.1 ± 2.5 were assessed for gait analysis during single-task and dual-task conditions. The participants’ sleep quality was assessed using the Pittsburgh Sleep Quality Index (PSQI) and gait was assessed using the BTS Gaitlab System. The participants were asked to walk at natural speed as a single-task condition, followed by walking while performing a cognitive task as a dual-task condition. The parameters assessed included the gait velocity (m/s), cadence (steps/min), step width (m), and stride length (m). The dual-task cost (DTC) on each gait parameter was calculated. The Mann–Whitney U test was used to compare the differences in the DTC on gait variables between the good and poor sleep quality groups and the Spearman correlation test was used to assess the correlation between total PSQI scores and the DTC. **Results**: At a significance level of *p* < 0.05, a significant difference in cadence between the two sleep quality groups was observed, in addition to a positive correlation between sleep quality and the DTC effect on gait mean velocity, cadence, and stride length. Our findings also revealed a greater DTC in participants with poorer sleep quality. **Conclusions**: These findings contribute to our perception of the significance of sleep quality in gait performance while multitasking in younger populations.

## 1. Introduction

Sleep quality plays an essential role in multiple physical, behavioral, and cognitive functions [1,2,3]. An individual’s sleep quality can be described as satisfaction with all aspects of their sleep experience and is usually defined by four factors: sleep efficiency, sleep latency, sleep duration, and wakefulness following sleep start [3]. Universal guidelines have been debated regarding the appropriate sleep duration for young adults [4]. Through a consensus developed by the American Academy of Sleep Medicine (AASM) and the Sleep Research Society (SRS), it has been recommended that young adults need more than nine hours of sleep per night [5], whereas the National Sleep Foundation in the US has released an updated recommendation stating that seven to eight hours of sleep is sufficient for the proper execution of daily activities [6,7]. Recent research in the Middle East showed that most young adults have shorter sleep durations than the recommended sleep guidelines [8,9]. Additionally, the literature reports that 40% of young adults suffer from poor sleep quality worldwide [10].

Investigations of sleep quality’s effect on cognitive and motor functions have recently drawn more attention regarding various populations from different age groups [11,12,13]. According to several findings, poorer sleep quality leads to greater impairments in younger adults in reaction time, performance lapses, and frequency of unintentional sleep episodes when compared with older adults [14]. Generally, poor sleep quality affects performance during simple tasks and results in a decline in the level of attention while increasing the attentional effort required [11,15]. Moreover, sleep deprivation tends to reduce attentional capacity and vary arousal levels, leading to difficulty in sustaining effort and multitasking performance [11]. 

Generally, multitasking is usually performed during a physical activity such as gait [16]. Gait is a complex rhythmic movement achieved through the association of multiple body systems [17]. The cerebellum and basal ganglia play important roles in the cognitive and automatic processes required for gait control through connections with the cerebral cortex and brainstem, respectively [18]. Studies have shown that low-quality sleep is linked to lower metabolic activity in the thalamic–basal ganglia circuit, the limbic and prefrontal regions, and the cerebellum [19]. Thus, such disruptions might alter gait control, resulting in gait disturbances and, eventually, falls [18]. 

Gait assessment has been a topic of interest over the past century [20]. Research has entailed utilizing various assessment tools and observational outcome measures to assess gait characteristics [20]. One emerging technological tool is the BTS GAITLAB system [21,22]. The use of the BTS GAITLAB has been validated in assessing gait spatiotemporal parameters in healthy children and adults [21,22]. 

Additionally, the effect of sleep quality on gait has been investigated in diseased populations, including elderly people with Parkinson’s disease [23]. Step width variability increases in patients with Parkinson’s disease who suffer from poor sleep quality [23]. The association between gait and sleep has also been investigated among community-dwelling elderly people given the expected decline in both sleep quality and alterations in gait patterns [24]. The literature has little information regarding the effects of sleep quality on gait in younger populations. However, it is known that sleep disorders begin at a younger age because of rapid lifestyle changes and growing responsibilities, including university and work [25,26]. 

Furthermore, gait control requires an adequate balance between the automatic and executive mechanisms [24]. An effective and common way to distinguish between these two mechanisms is by adding a dual task [27]. A dual task is an individual’s ability to perform two tasks at the same time in which they are required to coordinate their attention between both tasks while performing them simultaneously [28]. Usually, a decline in the performance of at least one of the tasks is expected while dual-tasking because of the capacity sharing theory, which proposes that when limited cognitive resources are shared simultaneously, a decrease in the efficiency of either task will be observed [29]. This performance decrement is known as the dual-task cost (DTC) [30]. 

Evidence suggests that sleep-quality-related disorders might influence task performance or motor response while implementing dual tasks [31]. The literature shows that an individual’s ability to divide attention while multitasking declines when exposed to sleep deprivation and adopting a poor sleep schedule [11]. Previous research has investigated the effect of sleep quality on postural control during a dual task on healthy young adults, where sleep quality was assessed using the Pittsburgh Sleep Quality Index (PSQI) [27]. Participants were divided into two sleep quality groups according to their total PSQI score and postural control was assessed while performing an arithmetic or a memory cognitive dual task [27]. Correspondingly, we hypothesized that young adults with poorer sleep quality would also demonstrate a greater decline in gait performance following the addition of a dual task. 

There is a lack of literature regarding the association between sleep quality and the dual-task cost on gait spatiotemporal parameters in younger populations. Therefore, we investigated the association between sleep quality and gait spatiotemporal parameters following the addition of a dual task to young adults.

## 2. Materials and Methods

### 2.1. Study Design, Participants, and Setting

This cross-sectional observational study was conducted at the Physiotherapy Research Laboratory at the University of Sharjah between May 2023 and October 2023. A sample of 65 healthy young adults (50.8% females and 49.2% males) with a mean age of 21.1 ± 2.5 volunteered to participate in the study and signed an informed consent form. All participants were assessed between 12 pm and 5 pm on weekdays between Monday and Thursday. The inclusion criterion was participants aged between 18 to 25 years old. Undergraduate and graduate students were recruited from a university setting through a series of in-classroom announcements. The exclusion criteria were as follows: (1) being athletic or adopting a lifestyle with high-interval training; (2) any recent major surgeries performed within the previous 2 months; (3) any existing musculoskeletal, neurological, or cardiorespiratory disorders; (4) current intake of medications with side effects interfering with concentration and balance; (5) any visual impairments or unmanaged visual myopia, farsightedness, or astigmatism; (6) any vestibular or middle-ear disorders; (7) any cognitive impairments or sleep disorders; (8) any sensory impairment; and (9) any use of sleep medications. 

### 2.2. Sample Size

Power analysis for correlation was performed with G-Power (version 3.1.9.7) to determine a sufficient sample size. For α = 0.05, two-tailed; a power of 0.8; and an effect size of 0.05, a sample of at least 29 participants was required.

### 2.3. Ethical Considerations

This study was approved by the Research Institute of Medical and Health Sciences of the University of Sharjah (reference number: REC-23-02-23-04-PG). All participants were educated about the study’s aims and procedure and a written informed consent form was acquired from each participant before the evaluation. 

### 2.4. Study Tools and Outcome Measures

#### 2.4.1. Sleep Assessment Questionnaire

The Pittsburgh Sleep Quality Index (PSQI) was used to assess sleep quality. The PSQI has been routinely used in clinical practice for the assessment of sleep quality and sleep disorders. It has been found that the PSQI has a specificity of 86.5% and a sensitivity of 89.6% in differentiating between individuals with good and poor sleep quality [32]. Previous research has revealed a moderate to strong test–retest reliability of the PSQI global score among healthy adolescents and young adults [32]. The PSQI distinguishes between sleep quality types to form two sleep groups for comparison. This differentiation was achieved by measuring seven domains: (1) subjective sleep quality, (2) sleep latency, (3) duration of sleep, (4) efficiency of sleep, (5) sleep disturbances, (6) the use of any sleep medication, and (7) daytime behavioral dysfunction [33]. A net score equal to or less than five indicated generally good sleep quality, while scores higher than 5 indicated generally poor quality of sleep [33].

#### 2.4.2. Baseline Cognitive Assessment

The baseline evaluation of the cognitive function of the participants was assessed using the short–oriented–memory–concentration test (SOMCT CogTest). The SOMCT CogTest has a test–retest reliability of 0.77–0.83 [34] and is used to assess cognitive areas responsible for orientation, concentration, and memory [35]. This test includes six questions and a total score of 28, where higher scores indicate better cognitive function [35].

#### 2.4.3. BTS GAITLAB System

Gait spatiotemporal parameters were assessed using the BTS GAITLAB system. The BTS GAITLAB system provides optoelectronic data about gait using eight high-frequency infrared cameras (BTS SMART-DX EVO) that can detect reflective markers placed on the participant’s landmarks according to the desired protocol, two RGB video cameras for recording, and a kit reflective marker [36,37]. The spatiotemporal parameters assessed included the gait velocity, cadence, step width, and stride length.

### 2.5. Study Procedure

Participants were asked to complete a demographics data sheet and the PSQI questionnaire. Subsequently, the participants were divided into two groups based on their PSQI scores: a good sleep quality group and a poor sleep quality group) [27]. Following this categorization, the participants underwent the baseline cognitive evaluation to assess their initial cognitive function using the (SOMCT CogTest). Before the gait analysis, all anthropometric data were acquired using the smart capture software in BTS GAITLAB [38]. Markers were then placed by a professionally trained research assistant on the participants’ landmarks according to anatomical points defined by the “Helen Hayes with Medial Markers” protocol [39]. Specifically, the protocol uses 22 reflective markers placed on the following landmarks: (1) the 7th cervical vertebra; (2) the right acromion; (3) the left acromion; (4) the 2nd sacral vertebra; (5) both anterior superior iliac spines (ASIS); (6) both greater trochanters; (7) both medial femoral epicondyles; (8) both lateral femoral epicondyles; (9) both shanks (approximately in the region of the fibular head); (10) both lateral malleoli; (11) both medial malleoli; (12) both calcaneus; and (13) both the 2nd and 3rd toe junctions [39].

Following the preparatory procedures, the participants underwent a 3D gait analysis in which they were instructed to walk at their normal speed along a 10-m walkway as a single task. The procedure was repeated twice; the first trial was used to familiarize the participants with the study method and the second trial was included in the final data processing. The participants were then asked to walk according to the same instructions while simultaneously performing a cognitive dual task, which involved spelling a five-letter word backward [40]. Data were processed using the assigned BTS software (Smart Analyzer, BTS Bioengineering, Milan, Italy) [36].

### 2.6. Statistical Analysis

The normality of the data was checked using the Shapiro–Wilk test and the descriptive variables are presented using mean ± standard deviation (SD). The DTC was calculated as the percentage of performance changes in gait parameters (gait velocity, cadence, step width, and bilateral stride length) under the two conditions (single-task and dual-task) using the following equation: {Dual Tasks − Single Tasks/Single Tasks × (100)} [41]. Positive results indicated a reduction in gait parameter performance after the addition of a dual task, while a negative result indicated improved performance while dual-tasking [41]. The data were not normally distributed; thus, the Mann–Whitney U test was used to compare the differences in the DTC on gait variables between the two sleep quality groups (good sleep quality and poor sleep quality). Spearman rho correlation was used to correlate sleep quality and DTC on gait variables. Statistical analyses were performed using IBM-SPSS 22.0 (IBM Corp., Armonk, NY, USA); the significance level was set at *p* < 0.05 and 95% confidence intervals were obtained.

## 3. Results

### 3.1. Demographics

This study included 65 homogenous participants with a mean body weight of 71.8 ± 15.2 kg and a mean height of 169.15 ± 8.9 cm. The total PSQI mean score was 6.46 ± 3.15, indicating generally poor sleep quality among the participants. Additional participant characteristics and baseline gait variables are shown in Table 1.

### 3.2. The Dual-Task Cost on Gait Variables and the Difference between Sleep Quality Groups

**Figure 1:**
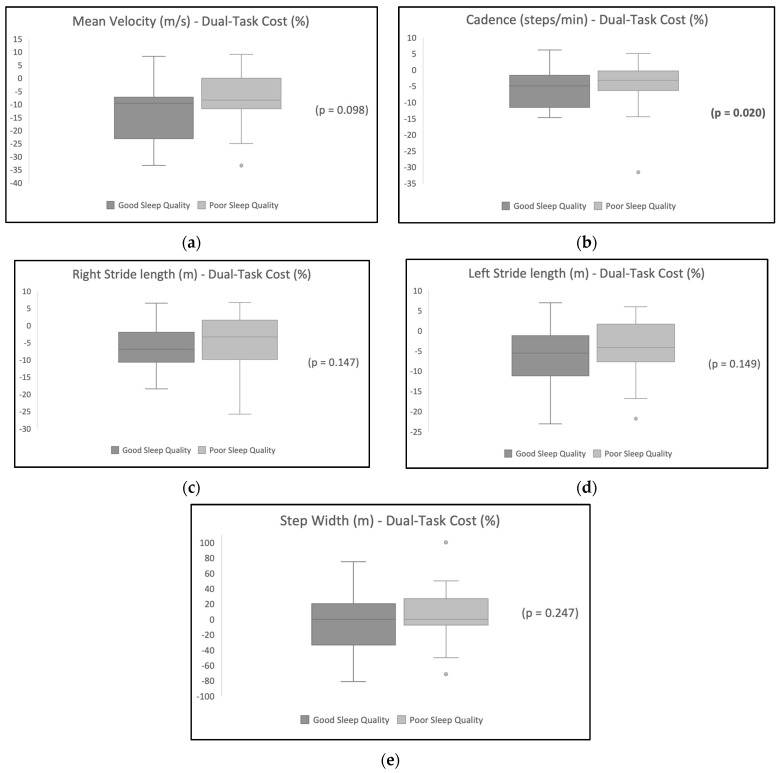
This figure shows the difference in DTC on each gait variable between the sleep quality groups. (**a**) Difference in the distribution of the effect of DTC on mean velocity between the good sleep quality group and poor sleep quality group; (**b**) difference in the distribution of DTC’s effect on cadence between the good sleep quality group and the poor sleep quality group; (**c**) difference in the distribution of DTC’s effect on right lower limb stride length between the good sleep quality group and the poor sleep quality group; (**d**) difference in the distribution of DTC’s effect on left lower limb stride length between the good sleep quality group and the poor sleep quality group; and (**e**) difference in the distribution of DTC’s effect on step width between the good sleep quality group and the poor sleep quality group.

Table 2 shows the DTC on gait variables and the results show a higher DTC for the participants with poor sleep quality. Table 3 shows the difference in the DTC between the sleep quality groups. Cadence was significantly different in the good sleep quality group compared with the poor sleep quality group (*p* < 0.05) after the addition of a dual task. Other variables were not statistically different between the two groups. Figure 1 illustrates the distribution of the DTC’s effect on each gait variable for participants with poor sleep quality in comparison with participants with good sleep quality.

### 3.3. Correlation between Sleep Quality and the Dual-Task Cost on Gait Variables

Table 4 shows the variables’ correlation coefficients between the total PSQI score and the DTC on gait variables of the total sample. The analysis shows a positive significant correlation between sleep quality and the DTC on gait mean velocity, cadence, and right and left lower limb stride lengths. However, there were no significant correlations between sleep quality and the DTC on step width (*p* > 0.05).

## 4. Discussion

This study investigated the association between sleep quality and gait spatiotemporal parameters following the addition of a dual task in young adults in the UAE. We hypothesized a decline in gait performance following the addition of a dual task, with the most prominent decrements observed in participants with poorer sleep quality. Our findings partially supported our hypothesis by revealing that the DTC had a greater effect on participants with poorer sleep quality; however, those with better sleep quality exhibited a lower DTC effect on gait, indicating that dual-tasking does not directly affect gait performance if the participant has relatively good sleep quality. Specifically, our analysis showed a significant difference in cadence between the two sleep quality groups. Moreover, we found a positive correlation between sleep quality and DTC on gait parameters, including mean velocity, cadence, and stride length. These significant correlations emphasize the growing body of research showing the effects of sleep quality on multitasking while walking.

We hypothesized that adding a dual task would have detrimental effects on gait spatiotemporal parameters because of capacity sharing theory [29]. However, our results showed that velocity, cadence, and stride lengths improved in the total sample, regardless of the participant’s sleep quality, following the addition of a dual task. These results are in line with a previous cohort that also noted improved performance when dual-tasking was combined with walking [42]. One possible explanation is that the dual task provided may not have sufficiently challenged the participants’ cognitive resources, as walking is an automatic motor task that typically requires minimal use of attention-demanding executive control centers [43]. Moreover, adding a cognitive task could have shifted attention from motor control related to walking, thereby enhancing the consistency and automaticity of the walking patterns in response [44]. An increase in speed while performing a concurrent dual task with walking was also observed in a previous study in which individuals showed an increase in gait velocity while carrying shopping bags in a simulated setting [45]. The authors hypothesized that paying more attention to a concurrent task would result in a more spontaneous pattern leading to walking at higher speeds [45].

On the other hand, our results showed that the dual task addition had a negative effect on step width. Step width for most participants decreased following the addition of a dual task. Wider step width has also been shown to improve lateral stability during walking, leading to a steadier gait [46]; thus, a narrowed step width may decrease the base of support and postural stability. Previous research has shown that increased step width is associated with a decrease in the mediolateral local and orbital stability of the C7 vertebra, indicating a decline in the stability and consistency of mediolateral trunk motion [46]. Similar recent results showed a decreased step width during a typical conversational dual task [47].

Furthermore, our analysis shows a significant difference in the DTC on cadence between the good and poor sleep quality groups. The literature shows that adding a dual task has a negative impact on gait spatiotemporal parameters, including cadence, among healthy and diseased populations [48]. The addition of a dual task to walking among older community-dwelling individuals has a negative effect on gait speed, cadence, and stride length [48]. Generally, previous research has focused only on studying the effects of adding cognitive and motor dual tasks on gait control [17,30,31,44,45,47,48,49]. However, the results of the present study provide a unique contribution to the literature as there have been no investigations comparing the DTC on gait spatiotemporal parameters between good and poor sleep qualities among younger populations.

Supporting the increased DTC in the poor sleep quality group, our investigation shows a positive correlation between the DTC on gait mean velocity, cadence, and bilateral stride lengths and the total PSQI score. This can be interpreted as a decline in mean velocity, cadence, and bilateral stride lengths after adding a dual task in individuals with poor sleep quality. These results are similar to those of a previous study that investigated the effect of sleep deprivation on divided attention performance in 30 healthy males exposed to 40 h of continuous sleep deprivation [11]. The results of this study showed that the more individuals that were deprived of sleep, the more performance deteriorated performance during tasks [11]. Sleep quality’s effect on cognitive function has also been studied in preterm and full-term children and it was found that the poorer the child’s sleep quality, the higher the number of awakenings after sleep onset and the more reduced dual-task performance was [50].

Our study also shows that 56.9% of the participants had poor sleep quality. Similar results have been reported multiple times over the previous decades [51,52]. The literature also reports that poor sleep quality is associated more with females than with males, with a higher incidence of nighttime wakefulness and reduced sleep duration and efficiency [53,54]. These findings are aligned with our results, in which females comprised 62.6% of the poor sleep quality group.

According to our knowledge, studies have previously emphasized the importance of sleep quality in the efficiency of multiple biological and cognitive functions; however, there are few health recommendations and promotional campaigns regarding the importance of improving sleep quality in young adults [55]. Thus, the results of our studies provide evidence for the significance of implementing public educational programs on the importance of improving sleep quality to ensure better physical and cognitive performance.

### Limitations of the Study

This study had a few limitations that justify further discussion and that should be addressed in future works. Because all participants had a higher education level, our results cannot be generalized to populations with lower educational levels. Additionally, as the poor sleep quality group was mostly females, further research thoroughly investigating each gender would therefore be recommended. Furthermore, although we used a valid and reliable subjective sleep assessment tool, future studies with more objective sleep assessments are necessary to further investigate the effects of sleep quality, such as the duration of each sleep cycle phase. Further recommendations include adding a triple-task to challenge neural resources since the population consisted mostly of young healthy educated adults.

## 5. Conclusions

We identified statistically significant associations between poor sleep quality and reductions in gait performance during the addition of a dual task. Our results show that studies of sleep quality effects should be initiated at a younger age as the percentage of sleep-related issues has recently increased in this population [12,26]. Furthermore, future studies with more objective sleep assessment tools are recommended to further understand the association between specific sleep disorders and changes in performance.

## Figures and Tables

**Table 1:** Demographic characteristics of the participants with gait variables.

Demographic Characteristics	Good Sleep Quality Group (N = 28) Mean ± SD	Poor Sleep Quality Group (N = 37) Mean ± SD	*p*-Value
Age (years)	20.89 ± 1.89	21.16 ± 2.98	0.888
Gender (females, males) +	28 (10, 18) +	37 (23, 14) +	0.036
Weight (kg)	74.13 ± 16.1	70.04 ± 14.48	0.263
Height (cm)	171.07 ± 9.64	167.70 ± 8.26	0.114
BMI (kg/m^2^)	25.17 ± 4.43	24.84 ± 4.50	0.629
PSQI (score out of 21)	3.61 ± 1.26	8.86 ± 2.44	0.000
SOMCT (score out of 28)	27.04 ± 1.07	26.81 ± 1.10	0.397
Mean velocity (m/s)	1.21 ± 0.13	1.22 ± 0.13	0.945
Cadence (steps/min)	112.29 ± 7.99	112.27 ± 7.54	0.952
Step width (m)	0.11 ± 0.14	0.08 ± 0.03	0.816
Right Stride length (m)	1.30 ± 0.10	1.31 ± 0.12	0.942
Left Stride length (m)	1.30 ± 0.08	1.31 ± 0.12	0.586

+ Number. BMI, body mass index; PSQI, Pittsburgh Sleep Quality Index; SD, standard deviation; SOMCT, short orientation–memory–concentration test.

**Table 2:** The effect of dual-task cost (%) on gait variables.

Variable	DTC Total Sample (N = 65) (Mean ± SD)	DTC Good Sleep Quality (N = 28) (Mean ± SD)	DTC Poor Sleep Quality (N = 37) (Mean ± SD)
Mean velocity (m/s)	−10.01 ± 10.04	−12.87 ± 10.96	−6.74 ± 11.15
Cadence (steps/min)	−4.30 ± 4.93	−6.15 ± 5.79	−3.91 ± 6.22
Step width (m)	4.09 ± 38.54	−3.67 ± 42.42	9.89 ± 34.71
Right Stride length (m)	−5.53 ± 7.13	−6.97 ± 6.71	−4.33 ± 7.33
Left Stride length (m)	−5.26 ± 6.79	−6.75 ± 6.96	−1.60 ± 16.89

DTC, dual-task cost. Note: Dual-task costs reflect performance variations as a percent of each participant’s single-task performance. Positive values indicate declines in performance under dual-task conditions, while negative values indicate improvements.

**Table 3:** Differences in DTC between the sleep quality groups.

Variable	Mann–Whitney U	*p*-Value
Mean velocity	394.0	0.098
Cadence (steps/min)	342.5	0.020 *
Step width (m)	431.0	0.247
Right stride length (m)	408.5	0.147
Left stride length (m)	409.0	0.149

Mann–Whitney U test of dual-task cost on gait variables between the two sleep quality groups. Bold value with * indicates significance at *p* < 0.05.

**Table 4:** Correlation between sleep quality and dual-task cost on gait variables.

Variable	Spearman Rho Correlation	*p*-Value
Mean velocity	0.373	0.002 *
Cadence (steps/min)	0.407	0.001 *
Step width (m)	−0.037	0.772
Right stride length (m)	0.278	0.025 *
Left stride length (m)	0.296	0.017 *

Spearman rho correlation test between total PSQI score and dual-task cost on gait variables for all the participants. Bold values with * indicate significance at *p* < 0.05.

## Data Availability

The datasets analyzed in the current study are available from the corresponding authors upon reasonable request.
